# Dynamic Analysis and Intelligent Control Strategy for the Internal Thermal Control Fluid Loop of Scientific Experimental Racks in Space Stations

**DOI:** 10.3390/e22010072

**Published:** 2020-01-06

**Authors:** Ben-Yuan Cai, Hui-Yi Wei, Yun-Ze Li, Yuan-Yuan Lou, Tong Li

**Affiliations:** 1 School of Aeronautic Science and Engineering, Beihang University, Beijing 100191, China; caibenyuan@buaa.edu.cn (B.-Y.C.); weihuiyi27@163.com (H.-Y.W.); carol.1230@163.com (Y.-Y.L.); 2 Institute of Engineering Thermophysics, North China University of Water Resources and Electric Power, Zhengzhou 450045, China; 3 Advanced Research Center of Thermal and New Energy Technologies, Xingtai Polytechnic College, Xingtai 054035, China; 4 Chengyi Academy of PKUHS, Peking University, Beijing 100080, China; litong@i.pkuschool.edu.cn

**Keywords:** fuzzy incremental control, scientific experimental rack, thermal control system, space station

## Abstract

Scientific experimental racks are an indispensable supporter in space stations for experiments with regard to meeting different temperature and humidity requirements. The diversity of experiments brings enormous challenges to the thermal control system of racks. This paper presents an indirect coupling thermal control single-phase fluid loop system for scientific experimental racks, along with fuzzy incremental control strategies. A dynamic model of the thermal control system is built, and three control strategies for it, with different inputs and outputs, are simulated. A comparison of the calculated results showed that pump speed and outlet temperature of the cold plate branch are, respectively, the best choice for the control variable and controlled variable in the controller. It showed that an indirect coupling thermal control fluid loop system with a fuzzy incremental controller is feasible for the thermal control of scientific experimental racks in space stations.

## 1. Introduction

Space stations have become an indispensable experimental platform for human beings to operate long-term advanced scientific experiments upon in the frontier of space, which has a microgravity environment. The International Space Station (ISS) is humanity’s largest foothold in space [1]. From 2000, when the first astronauts boarded the ISS, to now, more than 2600 unique experiments have been conducted on the ISS during the past 18 years of continuous research [2]. The diversity of research means a diversity in the experiments’ environment control requirements and the difficulty of integration. At present, developing standard interface racks (SIRs) [3] is the best solution.

Standard interface racks are designed around the concept of providing a set of common interfaces for modular experiments [4]. Expedite the Processing of Experiments to Space Station (EXPRESS) racks are the most widely used SIR, and provide accommodation and facilitates operations for microgravity-based research payloads on the ISS [5]. There have been many space experiments conducted in EXPRESS racks and similar racks, such as the European Drawer Rack (EDR), which aids with biological experiments, human physiology and adaptation experiments, and physical science experiments [1]. The EXPRESS rack can accommodate eight single middeck lockers and two international subrack interface standard drawers, and in order to realize the thermal control of payloads, a defined Active Thermal Control System (ATCS) Kit has been developed. The ATCS consists of a set of cold plates, some with a heat exchanger, and it provides a mechanism for transporting the heat load generated by the experiments to Internal Thermal Control System (ITCS) loops [6]. The payloads are in parallel or series connection in the thermal control fluid loop system. Each payload rack is connected to the ITCS, which can supply cooling fluid at 15.6 to 18.3 °C [7] via a permanently installed kit by flex hose assemblies [6], and the racks are in parallel connection with each other. Generally, the coolant flow of the ITCS loops flows into the payloads in the rack directly, and the flowrate is regulated by a Rack Flow Control Assembly (RFCA), which includes a modulating valve, a flow sensor, and a temperature sensor. The RFCA is located on the outlet line of the payload rack to maintain either a specific flow rate or a specific outlet temperature as determined by the user [7], which can realize the thermal control of the rack overall. Owing to the fact that the RFCA cannot control the flowrate of the cold plate branches, the temperature control of experimental payloads requires other accessories, including thermoelectric coolers (TECs), heaters, and fans, etc. In a few applications a heat exchanger is mounted within the experiment facility rack. Each side of the heat exchanger accommodates only one single loop flow, with the ITCS side being designated the cold side and the experiment rack the hot side [6,8].

Research on the control strategy of thermal control systems in space stations has focused on ITCS loops, which consist of system flow control assemblies, three-way mixing valves (TWMV), rack flow control assemblies (RFCA), cold plates, pressure sensors, temperature sensors, pump bypass assemblies (PBA), and heat exchangers [9]. The control algorithm used in thermal control systems in space stations is mainly a traditional PID control algorithm [10,11,12,13,14]. In the Columbus module there are two pairs of mixing valves in the water loop cooling system which are controlled by PID regulators to hold temperature set points. Paper [15] has verified the stability of a control system based on control theory using the thermal software tool ESATAN/FHTS. Valenzano et al. have taken the gain of a control algorithm as a parameter to verify the stability of the control system of Node 2 and 3, with the dangerous conditions of space stations being predicted [16]. De Palo S et al. have analyzed the stability of the thermal and hydraulic control loop of the Columbus in the ISS by using the theory of automatic control. The stability of the PID algorithm and the system stability when the control parameters change were verified by comparison with ground experiment, simulation, and flight data [17]. The thermal control system in the Human Research Facility (HRF) experiment rack, which was developed by NASA, has three internal flowrate controllers that use a modulated solenoid valve, flow meter, and a dedicated PID controller [4].

Compared with the traditional thermal control fluid loop system described above, there are many novel thermal control designs that can be learned despite some of them not being targeted for space stations. Wei Guo et al. have proposed an adaptive thermal control cold plate module (TCCM) which can be used in scientific experimental racks. The TCCM could provide flow rate and temperature co-adjustments by using a Shape-Memory-Alloy (SMA) assembly, which possesses self-driven abilities [18]. S.H. Lee et al. have proposed a Hybrid Thermal Control System (H-TCS) in which different operational modes are considered, namely, single phase, two-phase, basic heat pump and heat pump with liquid-side, and suction-side heat exchanger. This thermal control system would satisfy the diverse thermal requirements of different space missions [19]. A novel hardware component called the Sublimator Driven Coldplate (SDC) has been developed under NASA’s Constellation Program. It targets equipment which has a low heat load, short transport distance, and short mission duration requirements, and which has the potential to replace an entire thermal control system with one hardware component [20]. Kim et al. have suggested a new spacecraft thermal control hardware system composed of two parallel channels working for a heat pipe (HP) and a solid–liquid phase change material (PCM) for a high heat dissipating component which works intermittently with short duty [21]. As can be seen, related thermal control designs focus on the application of new materials and two-phase systems, which need a great deal of verification for use the spacecraft.

Intelligent control has not been widely used in thermal control systems in space stations and relative studies are scarce. Expert control is simply used to coordinate multiple thermal control subsystems and manage the whole system under some special conditions in the ISS. However, there are studies which have addressed the thermal control of spacecraft, electronic equipment, and so on. Yingnan Cui et al. have proposed an adaptive fuzzy controller for thermal management of microprocessors and have conducted an experiment which demonstrates that the adaptive fuzzy controller maintains control quality when faced with severe variations of the thermal model [22]. Trevor Hocksun Kwan et al. have proposed a fuzzy logic controller (FLC) which aims at temperature control of fuel cell stacks. Their FLC integrates both a combined Thermoelectric Generator-Thermoelectric Cooler (TEG-TEC) control method and variable coolant rate techniques to achieve both active temperature control (in TEC mode) and energy harvesting capability (in TEG mode) of the thermoelectric device [23]. Yun-Ze Li et al. have presented a fuzzy coordination control strategy used in a microchannel-heat-exchanger (MHE) space cooling network for future spacecraft, and demonstrated that it has better performance than single-input PID controllers [24].

In order to achieve more efficient and stable thermal control of scientific experimental racks in space stations, an indirect coupling thermal control fluid loop system is proposed here which has an isolated fluid loop in the rack. Compared with the fluid loop system which connects the cooling fluid of the ITCS directly by flex hose assemblies, it adds an intermediate heat exchanger, a pump assembly, and a number of regulating valves. There are several advantages to the proposed system: (1) it allows the thermal control system in the rack to be designed without having to meet all requirements of the ITCS loops, which have many limitations; (2) any contamination originating in the experiment components or leakage from the secondary cooling loop will be confined at rack level and will not affect other racks; and (3) the temperature of the experimental payloads in the rack can be controlled precisely. A dynamic model of the thermal control system is also built using the software tool AMESim. Then, three intelligent control strategies for the thermal control system which employ two fuzzy incremental controllers are presented, and the difference among these is the input and output of the controller. Finally, a simulation of the three control strategies is performed. In addition, the results of the simulation are compared and analyzed in detail.

## 2. Controlled Object Description and the Intelligent Control Strategy

### 2.1. Indirect Coupling Thermal Control Fluid Loop System Description

**Figure 1:**
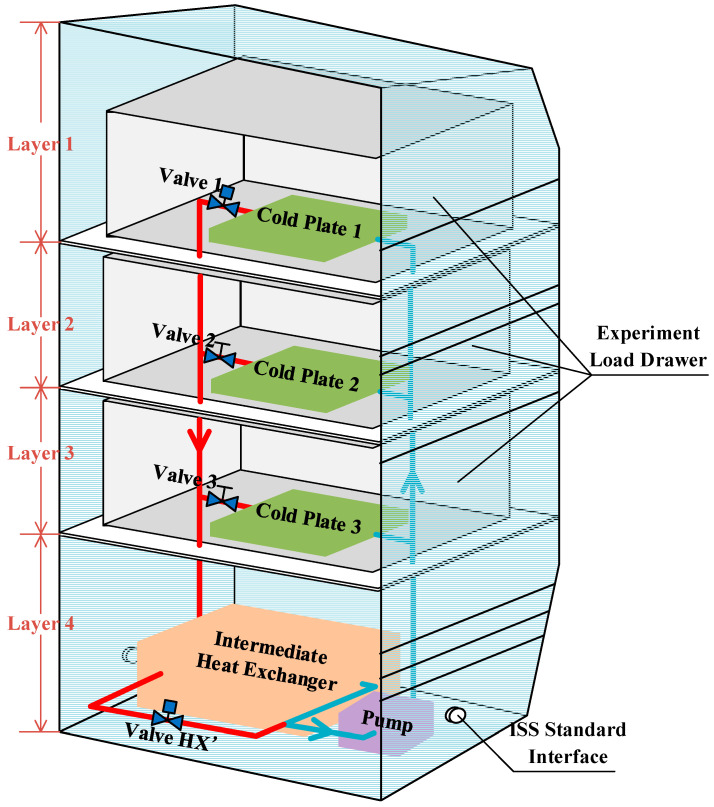
Layout of the scientific experimental rack. Legend: ISS, International Space Station; HX’, the bypass of heat exchanger.

**Figure 2:**
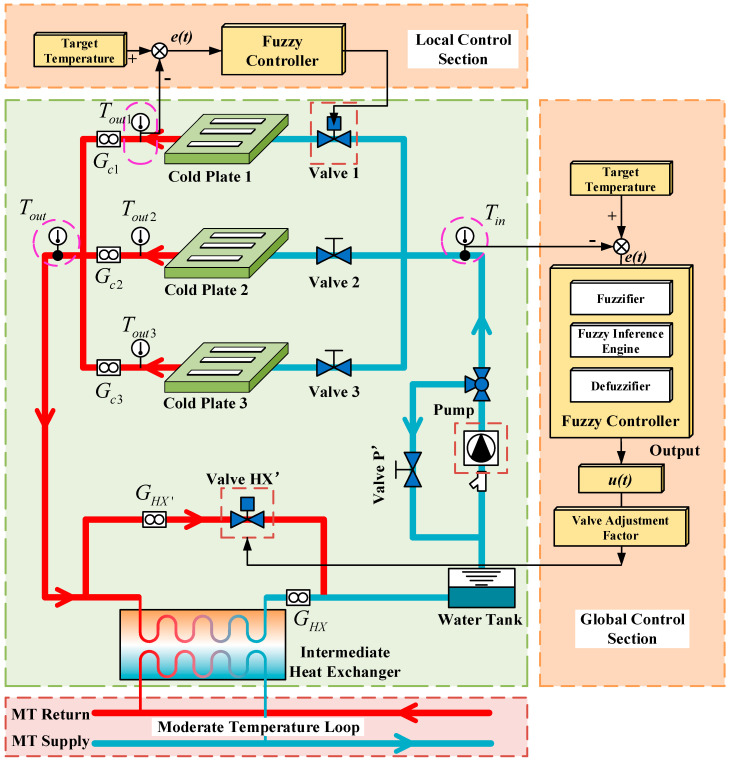
Schematic of the indirect coupling thermal control fluid loop system with control strategy. *T_in_* is inlet temperature of the cold plate branch; *T_out_*_1_, *T_out_*_2_ and *T_out_*_3_ are outlet temperatures of cold plate 1, 2 and 3; *T_out_* is outlet temperature of the cold plate branch; *G_c_*_1_, *G_c_*_2_ and *G_c_*_3_ are flowrates of the cold plate branches; *G_HX_* and *G_HX_*_’_ are flowrates of the heat exchanger and the heat exchanger bypass.

In this paper, an indirect coupling thermal control single-phase fluid loop system is constructed to keep experimental payloads within their thermal requirements in a scientific experimental rack. The layout of the thermal control loop in a scientific experimental rack is shown in Figure 1. Three experimental payloads drawers are arranged hierarchically in the rack, and different experiments can be carried out in them. In addition, there is a cold plate in each drawer to take waste heat away to ensure that the experiment is carried out well. Furthermore, there is an intermediate heat exchanger, a pump, and the like assembly in the bottom of the rack. The heat exchanger is the key component of the whole fluid loop system; it isolates the internal loop of the scientific experimental rack from the single-phase fluid loop of the internal thermal control system of the space station. A schematic of the thermal control loop is shown in Figure 2. There are two loop sections during the heat dissipation process: the secondary water loop (SWL) and the primary water loop (PWL). The former refers to an internal loop which provides cooling water to the experimental payloads in the rack, while the latter refers to a moderate temperature loop which routes the cooling fluid of the internal thermal control system of the space station to the intermediate heat exchanger of the SWL [8]. These two water loops are coupled by the intermediate heat exchanger. The primary water loop in the rack is equipped with a circulating pump, which drives cooling fluid starting from the water tank, passing it through the circulating pump, three parallel cold plates, and the intermediate heat exchanger, and finally returning it to the water tank. In this process, the waste heat is transferred from the experimental payloads to the cold plates in the way of heat conduction. The cooling water, after collecting waste heat from the cold plates by heat convection, passes through the intermediate heat exchanger, which transfers the waste heat to the primary water loop. Finally, the waste heat is rejected through the radiator of the space station by the primary water loop.

### 2.2. Intelligent Control Strategy

#### 2.2.1. Control Parameter Setting

The main objective of the thermal control system is to make each experimental payload within a suitable temperature range, and when the heat release of the experimental payload changes, that a corresponding adjustment can be made to ensure that the payload still works well. To meet the above requirements, each parallel cold plate branch is equipped with an electric regulating valve, and the intermediate heat exchanger has a bypass with an electric regulating valve. In theory, if the total inlet and outlet temperatures of the cold plates are controlled within a suitable range, the general temperature control requirement of the experimental payloads can be met. To cope with the potentially high cold plate temperature caused by an experimental payload which has a higher thermal load than others, the flowrates of each cold plate need further regulation. Thus, a control strategy of the internal thermal control fluid loop system is formed which is divided into two main hierarchies: the local hierarchy is used to adjust the valve opening in each cold plate branch $\varphi_{c,i}$ to regulate the flowrate in order to control the outlet temperature of each branch $T_{out,i}$, and the global hierarchy is used to adjust the valve opening in the bypass of the heat exchanger $\varphi_{HX\prime }$ or the pump speed n to control the total inlet/outlet temperature $T_{in}$/$T_{out}$. Control of the valves in each cold plate branch can cope with different thermal loads of each experimental payload, while control of the valve in the bypass of the heat exchanger or the pump speed can ensure that the flowrate and temperature of the fluid meets the heat exchange demand of the whole system and eliminate the flowrate disturbance among the cold plates as much as possible. As has been mentioned, the function of the local controller is local control and the function of the global controller is global control, so the implementation cycle of the local controller should be shorter than the global controller to achieve more precise temperature control of the cold plates. In this paper, the implementation cycle of the local controller is 10 s and for the local controller 20 s, namely, the valve in each cold plate branch responds every 10 s and the valve opening in the bypass of the heat exchanger or the pump speed responds every 20 s. In addition, it should be noted that the inlet and outlet temperatures indicate the cooling water temperature if there are no other explanations. The settings of control variables are showed in Table 1 and Table 2.

#### 2.2.2. Fuzzy Incremental Controller

**Figure 3:**
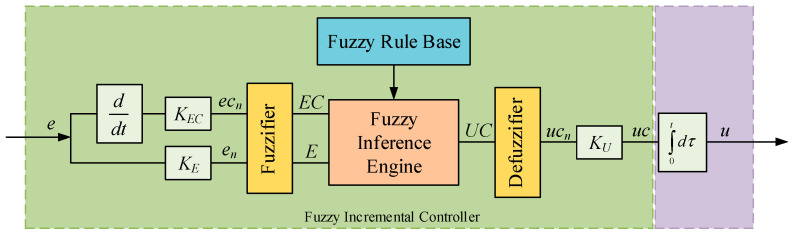
Structure of the fuzzy incremental controller.

In this paper, fuzzy incremental control is employed in the two controllers of the fluid loop system. Fuzzy incremental control is an improved control mode based on traditional fuzzy control, which is mainly aimed at practical problems such as the existence of steady state control residuals and the difficulty of controllers adapting to change in control object parameters [25]. Moreover, the output of the fuzzy control is the increment of the control variable, which can be the final control variable by integration. It can not only eliminate the error but also improve the reliability of the control algorithm. A structure chart of the fuzzy incremental controller is presented in Figure 3. It consists of four main parts, namely, the fuzzifier, fuzzy rule base, fuzzy inference engine, and defuzzifier. *K_E_* and *K_EC_* are the quantization factors of error and error change, respectively. *ec_n_* and *e_n_* are the quantized inputs. *E* and *EC* are the fuzzy sets of the inputs. *UC* is the fuzzy set of the output. *uc_n_* is a dimensionless output of the increment of the control variable. *K_U_* is the proportionality coefficient of the output. *uc* is the increment of the control variable. *u* is the control variable. As mentioned above, the input of the controller is the temperature error and the output can be the pump speed or the opening of the electric regulating valve.

First of all, we multiply the actual variables *e* and *ec* by quantization factors and transform them to the range of the universe [−1, 1].

(1) $$e_{n}=K_{E}\times e$$

(2) $$ec_{n}=K_{EC}\times ec$$

where *e* is the error of input and *ec* is the error change.

Then, we transform the input variables to fuzzy variables by the singleton fuzzifier method.

(3) $$\mu_{A}\left(x\right)=\{\begin{array}{c} 1\left(x=x_{0}\right)\\ 0\left(x\neq x_{0}\right) \end{array}$$

**Figure 4:**
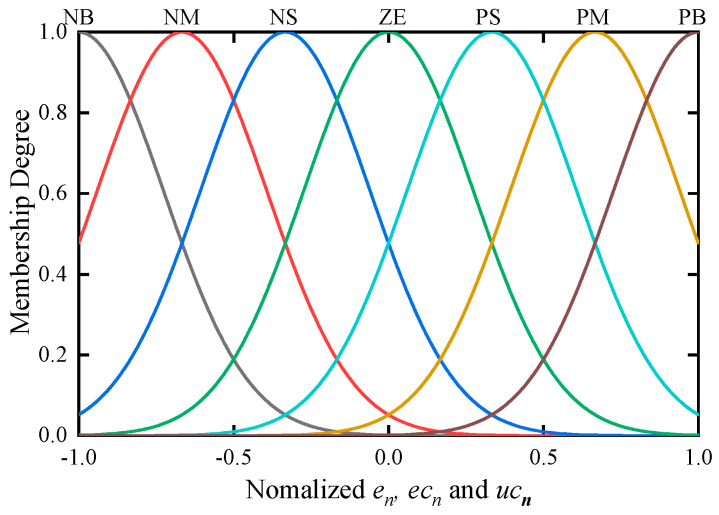
Membership functions of the input and output.

We then divide inputs and output into seven fuzzy sets, as shown in Table 3. Each fuzzy set is defined by a Gaussian membership function, as shown in Figure 4.

The fuzzy rule base is composed of a series of fuzzy conditional sentences in the following form:

*IF e_n_ is E_j_ and ec_n_ is EC_i_, THEN uc_n_ is UC_k(i,j)_*

Equation (4) can be used to generate fuzzy decision rules, as shown in Table 4.

(4) $$\left\{ \begin{array}{l} \begin{array}{c} k\left(i,j\right)=-\phi \times i-\left(1-\phi \right)\times j\\ \phi =0.8-0.1\times \left|i\right| \end{array}\\ uc_{R,k(i,j)}=\{\begin{array}{c} floor\left(k\right),k<0\\ ceil\left(k\right),k>0 \end{array} \end{array}\right.$$

where i,j∈{−3,−2,−1,0,1,2,3}. i, j and $uc_{R,k(i,j)}$ are, respectively, the fuzzy sets of the input and output. floor and ceil indicate the functions of round down and round up, respectively.

The principles of fuzzy rule setting are:

1. When the error *e* is negative, implying that the actual temperature exceeds the target temperature, the output of the controller should increase;
2. When the error *e* is positive, the output of the controller should decrease;
3. When the error *e* and *ec* are negative, the output of the controller should be increased; when the error *e* is negative and *ec* is positive, implying that the actual temperature is decreasing, the output of the controller should increase slightly or remain unchanged.
4. When the error *e* and *ec* are positive, the output of the controller should be decreased; when the error *e* is positive and *ec* is negative, implying that the actual temperature is increasing, the output of the controller should decrease slightly or remain unchanged.

The rule base has 49 rules. For the rule (*i,j*) ‘*IF E is A_i_ and EC is B_j_, THEN UC is C*_(*i,j*)_’, the implication relationship and membership function can be defined by Equations (5) and (6).

(5) $$R(i,j)=(A_{i}\times B_{j})\times C(i,j)$$

(6) $$\mu_{R(i,j)}(e_{n},ec_{n},uc_{n})=\mu_{E_{j}}(en)\wedge \mu_{EC_{i}}(ecn)\wedge \mu_{UC_{k(i.j)}}(uc_{n})$$

Because the rules in the rule base are juxtaposed, the operation rules of fuzzy relation and membership function contained in the whole rule base are

(7) $$R=U_{ij}R(i,j)$$

(8) $$\mu_{R}(e_{n},ec_{n},uc_{n})=\vee \left[\left(\mu_{A_{i}}(e_{n})\wedge \mu_{B_{i}}(ec_{n})\wedge \mu_{C_{\left(i,j\right)}}(uc_{n}\right)\right]$$

The Mamdani is adopted in the fuzzy inference engine, along with the operations of fetching maximum and minimum, and the fuzzy value of the output can be defined by Equation (9), i.e.,

(9) C=(A×B)∘R

where “×” is the Cartesian product, “∨” is the operation of the fetching maximum, “∧” is the operation of the fetching minimum, and “∘” is the compositional operation.

The purpose of defuzzification is to turn the fuzzy variables from fuzzy inference to clear variables. In this paper, the weighted average method is used and the formula for calculation is

(10) $$uc_{n}=\frac{\sum_{i=1}^{7}\sum_{j=1}^{7}uc_{R,k\left(i,j\right)}\mu_{k\left(i,j\right)}}{\sum_{i=1}^{7}\sum_{j=1}^{7}\mu_{k\left(i,j\right)}}$$

where $uc_{R,k\left(i,j\right)}$ indicates the rank of the fuzzy output corresponding to column *i* and row *j* in the rule table.

The control variable can be defined by Equation (11).

(11) $$u_{n}=u_{n-1}+K_{U}\times uc_{n}$$

### 2.3. Dynamic Modeling of Indirect Coupling Thermal Control Fluid Loop System

In this paper, the fluid flows in the fluid loop thermal control system can be understood by one-dimensional computations. LMS Imagine.Lab AMESim (Siemens PLM Software, Leuven, Belgium), which offers a complete 1D simulation suite with which to model and analyze thermal systems, etc., was employed for a simulation study. The models used for the simulation are described below.

#### 2.3.1. Model Development

##### Model of the Experimental Rack

The rack structure is the most basic model in the system. As mentioned above, three cold plates are arranged in layers in the rack, and each layer is isolated from the others. Hence, the rack is divided into four parts by layers, and each layer is modeled respectively. The specific structure of the rack is shown in Figure 1. Among the layers, the layer containing the heat exchanger, pump, and other components can be ignored because the heat exchange of these components and rack structure is very small compared to the cold plates, and can be neglected.

Taking layer 1 as an example, its dynamical property can be described by the differential equation

(12) $$m_{r,1}c_{r}\frac{dT_{r}}{d\tau }=q_{cr,1}+q_{ar,1}+q_{ar,2}-q_{r0,1}$$

(13) $$q_{cr,1}=\frac{A_{cr}}{R_{cr}}(T_{c,1}-T_{r,1})$$

(14) $$q_{r0,1}=A_{r0,1}h_{r0}\left(T_{r,1}-T_{0}\right)$$

where $m_{r,1}$ and $c_{r}$ are, respectively, the mass and specific heat capacity of the rack structure (layer 1). $q_{cr,1}$ is the heat exchange capacity of the cold plate with the rack structure and $q_{ar,1}$ indicates the heat exchange capacity between air in layer 1 and the rack structure. The parameter $q_{ar,2}$ indicates the heat exchange capacity between air in layer 2 and the rack structure, and $q_{r0,i}$ indicates the heat exchange capacity between the rack and the cabin air.

##### Model of the Cold Plates

Considering the fact that there is no essential difference between the temperature of the cold plates and experimental payloads in this study, experimental payload models are omitted and the thermal load is applied to the cold plate directly to simplify the calculation. Hence, the average temperature of the cold plate is focused on in the following analysis.

The dynamical property of the cold plate model can be described by the differential equation

(15) $$m_{c}c_{c}\frac{dT_{c,j}}{d\tau }=\Phi_{i}-q_{cw,i}-q_{cr,i}-q_{ca,i}$$

(16) $$q_{cw,i}=G_{c,i}c_{w}(T_{out,i}-T_{in})$$

(17) $$q_{cr,i}=\frac{A_{cr}}{R_{cr}}(T_{c,i}-T_{r,i})$$

(18) $$q_{ca,i}=A_{ca}h_{ca}(T_{c,i}-T_{a,i})$$

where $m_{c}$ and $c_{c}$ are, respectively, the mass and specific heat capacity of the cold plate. $\Phi_{i}$ is the thermal load. The parameter $q_{cw,i}$ characterizes the convection heat transfer between the cold plate and the cooling fluid, and $q_{cr,i}$ is the heat exchange capacity of the cold plate with the rack structure. $q_{ca}$ indicates the convection heat transfer between the cold plate and the air in layer *i*.

(19) $$q_{cw,i}=\frac{Nuk}{de}\cdot A_{cw}\eta_{0}\cdot \frac{T_{out,i}-T_{in}}{\ln\left[\left(T_{c,i}-T_{in}\right)/\left(T_{c,i}-T_{out,i}\right)\right]}=\alpha A_{cw}\eta_{0}\Delta t_{m,i}$$

The cold plates used in the thermal control system are tubular, so α=Nuk/de indicates the convection heat transfer coefficient of the tube surface. The variable k is the thermal conductivity of the cooling fluid. $\Delta t_{m,i}=\frac{T_{out,i}-T_{in}}{\ln\left[\left(T_{c,i}-T_{in}\right)/\left(T_{c,i}-T_{out,i}\right)\right]}$ is the logarithmic mean temperature difference and $T_{c}$ is the surface temperature of the cold plate. The parameter $A_{cw}$ indicates the total surface area involved in convective heat transfer. $\eta_{0}$ is the total efficiency of the cold plate and $\eta_{0}=1$ for the tube type cold plate without ribs in this system.

The calculation formula for the Reynolds number is

(20) Re=Gc,ideAcυ

where de and $A_{c}$ are, respectively, the equivalent diameter and cross section area of the tube in the cold plate. $G_{c,i}$ and υ indicate the mass flow rates and viscosity of the cooling fluid, respectively.

When Re<2300, the cooling fluid is in a state of laminar and the Nusselt number is a constant, i.e.,

(21) Nu=4.36

When Re>2300, according to the Dittus-Boelter formula, the cooling fluid is in a state of turbulent flow, i.e.,

(22) Nu=0.023Re0.8Prn

where n=0.4 if the fluid is heated and n=0.3 if it is cooled.

##### Model of the Intermediate Heat Exchanger

The effectiveness number of heat transfer units method (ε−NTU) is used to calculate the heat transfer of the heat exchanger. The concrete computation process is given in [26]. It is important to point out that a counter flow heat exchanger is adopted in this paper and that the steady state effectiveness relation is written as

(23) $$\varepsilon_{steady}=\frac{1-e^{-NTU(1-C_{r})}}{1-C_{r}\cdot e^{-NTU(1-C_{r})}}$$

where NTU is the number of heat transfer units and $C_{r}$ is the flow stream capacity ratio.

When calculating NTU, the overall heat transfer coefficient *U* needs to be calculated first, i.e.,

(24) $$U=\frac{1}{R_{1}+R_{wall}+R_{2}}$$

where *R*_1_ and *R*_2_ are the thermal resistance of the two fluids in the heat exchanger and *R_wall_* is the thermal resistance of the heat exchanging surface.

##### Model of the Pump

The main function of the circulating pump is to provide a steady flow of cooling water for the secondary water loop in the rack. The component model in AMESim can simulate the pressure and flow characteristics of the pump. Without considering the heat production and heat dissipation of the pump itself, the pump model provided by the software can be used directly. The parameters to be configured are the speed and displacement of the pump, and the total flowrate of the secondary water loop can be calculated by Equation (25), i.e.,

(25) $$G_{all}=q_{p}\times n_{p}$$

(26) $$G_{all}=G_{p\prime }+G_{c1}+G_{c2}+G_{c3}$$

where $q_{p}$ and $n_{p}$ are the displacement and speed of the pump, respectively, and $G_{p\prime }$ is the flowrate of the pump bypass.

##### Model of the Pipe

The heat exchange between the pipe and the surroundings can be ignored owing to the pipe’s being wrapped with thermal insulation material in practical applications. Thus, the pressure drop of the pipe is the only consideration used in modeling.

The pressure loss of the pipe is

(27) $$\left\{ \begin{array}{l} \Delta P=\sum \Delta p_{\lambda }+\sum \Delta p_{\zeta }\\ \sum \Delta p_{\lambda }=\sum \lambda \frac{L}{D}\left(\rho \frac{V^{2}}{2}\right)\\ \sum \Delta p_{\zeta }=\sum \zeta \left(\rho \frac{V^{2}}{2}\right) \end{array}\right.$$

where $\sum \Delta p_{\lambda }$ is the friction losses and λ is the Darcy friction factor. The parameters L and D indicate, respectively, the length and diameter of the pipe. $\sum \Delta p_{\zeta }$ is the minor losses and ζ is the minor losses coefficient. Additionally, ρ and V are the density and average flow velocity of the cooling fluid.

When Re<2300, the fluid is in a state of laminar flow, i.e.,

(28) λ=64/Re

When Re>2300, the fluid is in a state of turbulent flow, i.e.,

(29) 1λ1/2=−2.0log(ε/D3.7+2.51Reλ1/2)

where *D* is the pipe diameter.

In addition, the pressure losses of cold plates and some sensors can also be calculated by the pressure loss modeling given above.

#### 2.3.2. Definition of the Simulation Cases

The keystone of this paper was to contrast different control strategies which can be used in a thermal control loop inside experimental racks and to verify the effectiveness of fuzzy incremental control in a thermal control system. Through contrastive analysis, the optimal control strategy was obtained.

**Figure 5:**
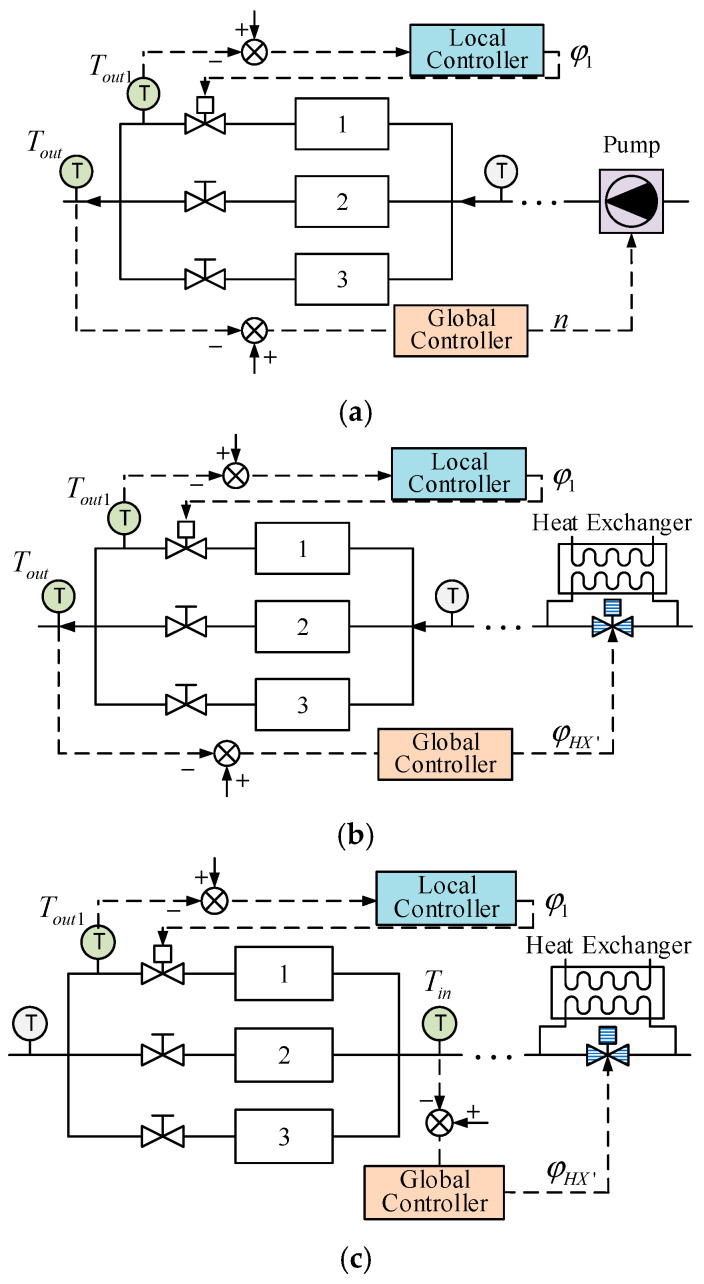
Three control strategies with different inputs and outputs. (**a**) The control variable is the pump speed and the controlled variable is $T_{out}$. (**b**) The control variable is the valve opening of the bypass of the heat exchanger and the controlled variable is $T_{in}$. (**c**) The control variable is the valve opening of the bypass of the heat exchanger and the controlled variable is $T_{out}$.

In this paper, three cases were simulated. As stated above, the cold plates are parallel, meaning they have the same status in the thermal control system. In order to simplify the simulation, it is assumed that the heating power of cold plate 1 $\Phi_{1}$ changes a lot throughout the simulating process while the heating powers of cold plates 2 and 3 are constant, meaning the controller is simply used in the cold plate 1 branch to realize more accurate control of the temperature. It is not only possible to study the validity of the local level of the control strategy but also the impact of the other two cold plates. The structure diagrams of the three control strategies are shown in Figure 5. The local level of the control strategy is the same; in this, the control variable is the valve opening of the cold plate 1 $\varphi_{c1}$ branch and the control variable is the outlet of cold plate 1 $T_{out1}$. The differences are shown in the Table 5 below.

It should be noted that the reason why the taking of the inlet temperature of the cold plate branch as the control target to adjust the pump speed is not adopted is that (1) when the pump speed is slow, the inlet temperature will be lower than the target temperature because the flowrate is very small but (2) when the pump speed is high, the inlet temperature will also be lower than the target temperature because the flowrate is big enough. That is to say, the system is uncontrollable using that strategy under those conditions.

In addition to the control strategy, there are no differences in the models and simulation parameters of the three cases. The main simulation parameters are shown in Table 6. The whole simulation process is divided into two stages based on the step time point of the heating power of cold plate 2, which is showed in Table 7.

**Figure 6:**
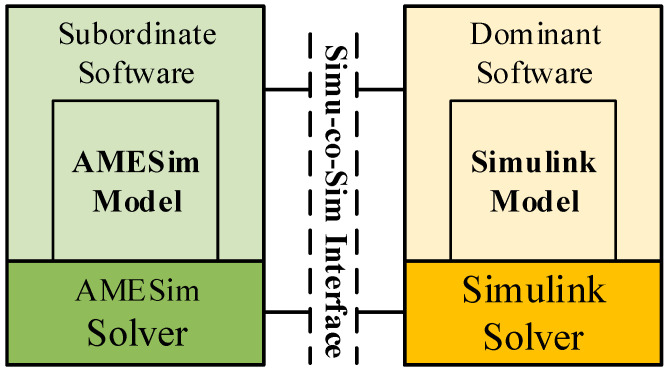
Data exchange and co-simulation process between AMESim and MATLAB/Simulink.

Although it is quite accurately efficient to build the models presented above in AMESim, the complex control algorithm is difficult to implement within it. In this paper, a co-simulation interface in AMESim was adopted to communicate with MATLAB/Simulink so that the control algorithm could be developed in Simulink. The specific data exchange and co-simulation process is showed in Figure 6.

## 3. Results and Discussion

In order to verify the effectiveness of the fuzzy incremental controller and reflect on its advantages, the fuzzy incremental controller is first compared with the PID. The two controllers are, respectively, employed in case 1, in which the controlled variable is the outlet temperature of the cold plate branch $T_{out}$ and the control variable is the pump speed n. The other simulation parameters are shown in Table 6.

**Figure 7:**
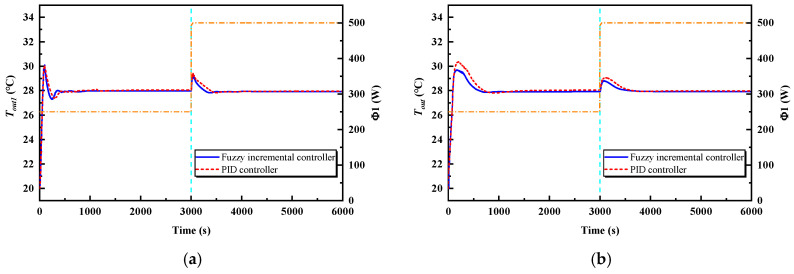
Temperature–time curves. (**a**) The outlet temperature of cold plate 1. (**b**) The outlet temperature of the cold branch.

The controlled variables of the local controller and global controller, as well as the outlet temperature of the cold plate 1 $T_{out1}$ and the outlet temperature of the cold branch $T_{out}$, are illustrated in Figure 7. The overshoots and settling times of $T_{out1}$ and $T_{out}$ with the two controllers are shown in Table 8. As can be seen, the overshoots of the outlet temperature of cold plate 1 $T_{out1}$ are almost the same as for the two controllers, but its settling times in the two stages with the fuzzy incremental controller are shorter than with the PID controller. As for the outlet temperature of the cold branch $T_{out}$, the overshoots in stage one are, respectively, 1.7 °C and 2.4 °C with the fuzzy incremental controller and the PID controller, and, in stage two, 0.8 °C and 1.1 °C. The settling times of the outlet temperature of the cold branch $T_{out}$ in the two stages with the fuzzy incremental controller are shorter than those with the PID controller, too. Taken together, the overshoots are smaller and the settling times are shorter for the controlled variables with the fuzzy incremental controller. In other words, the control effect of the fuzzy incremental controller is better than the PID controller in the thermal control system.

### 3.1. Case 1: The Controlled Variable Is $T_{out}$ and the Control Variable Is Pump Speed

In case 1, the bypass valve opening of the intermediate heat exchanger is constant. The controlled variable is the outlet temperature of the cold plate branch $T_{out}$, the setting temperature $T_{out,s}$ is 28 °C, and the control variable is the pump speed n.

**Figure 8:**
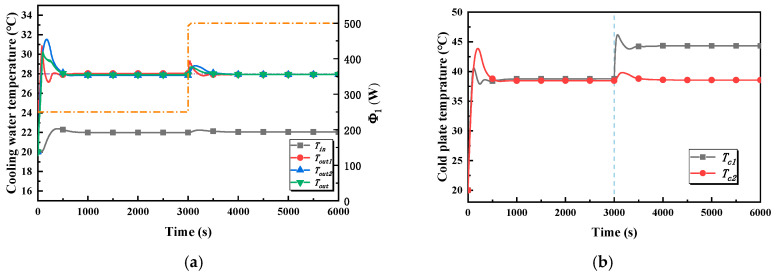
Temperature–time curves in case 1. (**a**) The inlet and outlet temperature of the cold branch and the outlet temperature of the cold in case 1. (**b**) The cold plate temperature in case 1.

The inlet and the outlet temperature of the cold branch and the outlet temperature of the cold plates are the main concerns, and are shown in Figure 8a. To be clear, owing to the fact that cold plate 2 and cold plate 3 are subjected to the same working conditions, only the relevant temperatures of cold plate 2 are shown in the figure. In stage one, all of the temperatures rise rapidly and tend towards stability after about 600 s under the action of the controller. The maximum value of the outlet temperature of the cold plate branch, which is the controlled variable in the global controller, is 30.2 °C, which means that the overshoot is 2.2 °C and the settling time is about 600 s. The maximum value of the outlet temperature of the two cold plates is 30.8 °C and 31.6 °C, respectively. However, it is obvious that the settling time of the outlet temperature of cold plate 1 is shorter, being about 300 s. The inlet temperature of the cold plate branch increases and stabilizes at 22 °C after 600 s. In stage two, the heating power of cold plate 1 jumps from 250 W to 500 W, which causes the outlet temperature of cold plate 1 to rise rapidly; its maximum is 29 °C, which means that the overshoot is 1 °C. The outlet temperature of cold plate 2 and the cold plate branch are increased, and the maxima are, respectively, 28.8 °C and 28.7 °C, which implies that the overshoots are within 1 °C. In addition, there is a slight increase in the inlet temperature of the cold plate branch, which is maintained at about 22 °C after 600 s.

Figure 8b illustrates the variation in cold plate temperature in case 1. In stage one, the highest temperatures of cold plate 1 and cold plate 2 are 40.3 °C and 43.9 °C, respectively. The temperature of cold plate 1 is stable after 350 s, but cold plate 2 reaches a stable temperature range after 500 s. The temperatures of the two cold plates are basically the same, being about 38.5 °C, in the steady state. In stage two, the temperature of cold plate 1 rises rapidly and finally stabilizes at 44.3 °C, while there is a slight rise in the temperature of cold plate 2.

**Figure 9:**
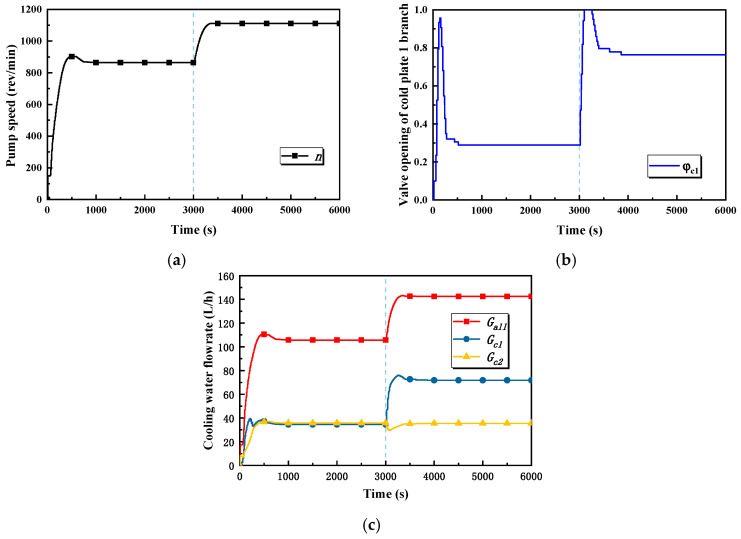
Pump speed, valve opening, and flowrate–time curves in case 1. (**a**) The pump speed in case 1. (**b**) The valve opening of the cold plate 1 branch in case 1. (**c**) Total flowrate and flowrates of the cold plate branches in case 1.

Main causes for the occurrence of the said phenomena can be explained by the variations in pump speed, the valve openings of cold plate branches, and the flowrate, which are shown in Figure 9. In stage one, the pump speed rapidly increases, which leads to an increase in the total flowrate of the loop. The valve opening of the cold plate 1 branch also rapidly increases, and with the effect of these two aspects, the flowrate of the cold plate 1 branch increases much faster than that of the cold plate 2 branch. As can be seen from Figure 9c, the flowrate of the cold plate 1 branch reaches a stable range after 200 s, but the cold plate 2 branch takes about 400 s to do so. Accordingly, the responses of cold plate 1’s relevant temperatures are faster than those of cold plate 2. In stage two, the valve opening of the cold plate 1 branch rapidly increases, resulting in a sharp increase in the flowrate of the cold plate 1 branch, which also leads to a decrease in the flowrate of the cold plate 2 branch. As a result, the temperature of cold plate 1 overshoots over a period of time.

### 3.2. Case 2: The Controlled Variable Is $T_{out}$ and the Control Variable Is $\varphi_{HX\prime }$

In case 2, the pump speed is constant and is set as 1000 rev/min. The controlled variable is the outlet temperature of the cold plate branch, the setting temperature $T_{out,s}$ is 28 °C, and the control variable is the bypass valve opening of the intermediate heat exchanger.

**Figure 10:**
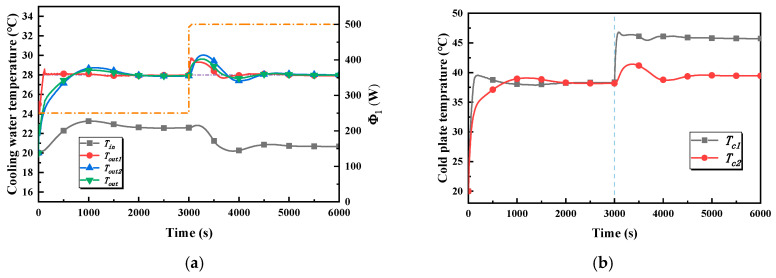
Temperature–time curves in case 2. (**a**) The inlet and outlet temperature of the cold branch and the outlet temperature of the cold in case 2. (**b**) The cold plate temperature in case 2.

The inlet and the outlet temperature of the cold plate branch and the outlet temperature of the cold plates are the main concerns of case 2, as is shown in Figure 10a. In stage one, all the temperatures rise rapidly and tend towards stability after about 2000 s under the action of the controller. The maximum value of the outlet temperature of the cold plate branch, which is the controlled variable in the global controller, is 28.5 °C, which means that the overshoot is tiny and is merely around 0.5 °C. The maximum values of the outlet temperature of the two cold plates are 28.5 °C and 28.7 °C, respectively. However, it is obvious that the settling time of the outlet temperature of cold plate 1 is shorter, being about 200 s. By contrast, cold plate 2, which is without the local controller, takes about 2000 s to get the same state. The inlet temperature of the cold plate branch is maintained at about 22.5 °C after 2000 s. In stage two, the heating power of cold plate 1 jumps from 250 W to 500 W, which causes the outlet temperature of cold plate 1 to rise rapidly; its maximum is 29.7 °C, which means its overshoot is 1.7 °C. The settling time is 1500 s. The outlet temperature of cold plate 2 and the cold plate branch increase and reach stability after 1500 s. Their maxima are, respectively, 30 °C and 29.6 °C. In addition, there is a decrease in the inlet temperature of the cold plate branch, which is maintained at about 20.7 °C after 1500 s.

Figure 10b illustrates the variation in the cold plate temperature in case 2. In stage one, the highest temperatures of cold plate 1 are 39.5 °C and 39 °C, respectively. The temperatures of the two cold plates are basically the same, being about 38.3 °C, in the stable state. In stage two, the temperature of cold plate 1 rises rapidly and is finally stabilized at 45.7 °C, and attains a stable state soon after. The highest temperature of cold plate 1 is 41.4 °C, and the temperature stabilizes at 39.4 °C after 1500 s.

**Figure 11:**
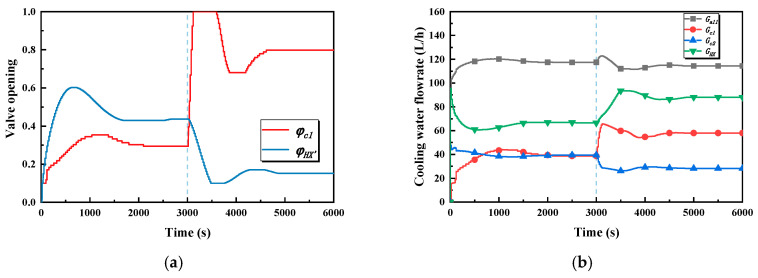
Valve opening and flowrate–time curves in case 2. (**a**) The valve openings of the cold plate 1 branch and the bypass of the heat exchanger in case 2. (**b**) Total flowrate and flowrates of the cold plate branches and the heat exchanger in case 2.

The main causes for the occurrence of the said phenomena in case 2 can be explained by the variations of the bypass valve opening of the intermediate heat exchanger, the valve openings of the cold plate branches, and the flowrate, which are shown in Figure 11. In stage one, the bypass valve opening of the heat exchanger increases because the outlet temperature of the cold plate branch is temporarily lower than the set temperature, which leads to a decrease in the flowrate through the hot side of the heat exchanger. The valve opening of the cold plate 1 branch increases, which leads to the flowrate of cold plate 1 branch increasing and for the flowrate of cold plate 2 branch to experience a relatively small decrease. That is to say, the change trends of the flowrate of the two plate branches are the opposite of one another. Hence, the outlet temperature of cold plate 1 and its temperature respond faster because the flowrate of the branch is small at the beginning. The flowrates of the two cold plate branches are, respectively, 38.7 kg/h and 39.4 kg/h after reaching a stable state result in the outlet temperature of the two cold plate branches, and the two cold plate temperatures are almost the same. In stage two, the valve opening of the cold plate 1 branch rapidly increases, resulting in a sharp increase in the flowrate of the cold plate 1 branch, which also leads to a decrease in the flowrate of the cold plate 2 branch. As a result, the temperature of the two cold plates are increased. Additionally, the bypass valve opening of the heat exchanger is decreased, rapidly bringing about an increase in the flowrate through the hot side of the heat exchanger, increasing the heat exchange between the SWL and the PWL.

### 3.3. Case 3: The Controlled Variable Is $T_{in}$ and the Control Variable Is $\varphi_{HX\prime }$

In case 3, the parameter settings are the same as in case 2, except for the fact that the controlled variable is the inlet temperature of the cold plate branch and that the setting temperature $T_{in,s}$ is 21 °C.

**Figure 12:**
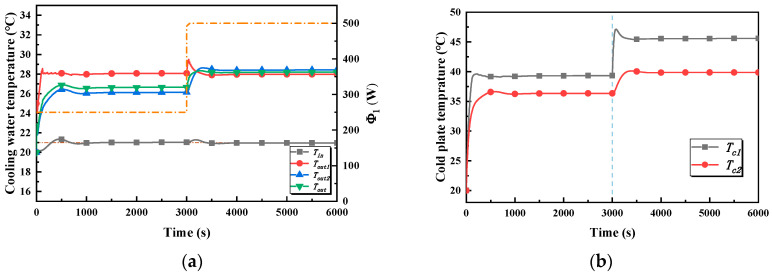
Temperature–time curves. (**a**) The inlet and outlet temperature of the cold branch and the outlet temperature of the cold in case 3. (**b**) The cold plate temperature in case 3.

The inlet and the outlet temperature of the cold plate branch and the outlet temperature of the cold plates are the main concerns of case 3, as is shown in Figure 12a. In stage one, all of the temperatures rise rapidly and tend towards stability after about 1000 s under the action of the controller. The maximum value of the inlet temperature of the cold plate branch, which is the controlled variable in the global controller, is 21.3 °C, which means that the overshoot is tiny, being merely around 0.3 °C. The maximum values of the outlet temperature of the two cold plates are 28.5 °C and 26.9 °C, respectively. However, it is obvious that the settling time of the outlet temperature of cold plate 1 is shorter, being about 300 s. Cold plate 2, which is the local controller, takes 1000 s to get the same state. Additionally, the outlet temperatures of cold plate 1 and cold plate 2 are, respectively, 28 °C and 26.1 °C, and there is a temperature difference of about 2 °C. In addition, there is an increase in the outlet temperature of the cold plate branch which is maintained at about 26.6 °C after about 1000 s. In stage two, the heating power of cold plate 1 jumps from 250 W to 500 W, which causes the outlet temperature of cold plate 1 to rise rapidly, and its maximumt is 29.3 °C. After 1000 s, it is maintained at about 27.9 °C. The outlet temperatures of cold plate 2 and the cold plate branch increase, and are, respectively, 28.4 and 28.2 °C in the stable state.

Figure 12b illustrates the variation in the cold plate temperature in case 3. In stage one, the temperature of cold plate 1 is stable after 200 s, but cold plate 2 reaches a stable temperature range after 750 s. The temperatures of the two cold plates are 39.3 °C and 36.3 °C, respectively, in the steady state. In stage two, the temperature of cold plate 1 rises rapidly and finally stabilizes at 45.5 °C, while the temperature of cold plate 2 finally stabilizes at 40 °C.

**Figure 13:**
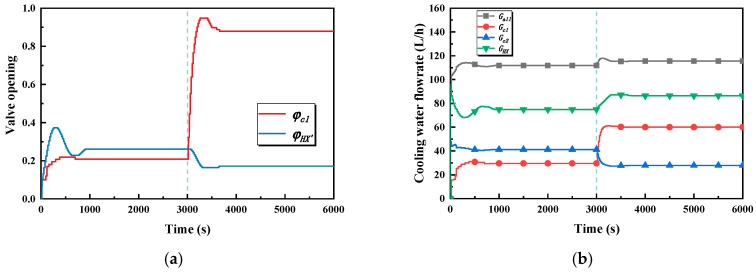
Valve opening and flowrate–time curves in case 3. (**a**) The valve openings of the cold plate 1 branch and the bypass of the heat exchanger in case 3. (**b**) Total flowrate and flowrates of cold plate branches and the heat exchanger in case 3.

The variations in the bypass valve opening of the intermediate heat exchanger, the valve openings of the cold plate branches, and the flowrate in case 3 are shown in Figure 13a. In stage one, the trend of the bypass valve opening of the heat exchanger and the valve opening of the cold plate 1 branch is as same as in case 1, but it is more responsive in case 3. The flowrates of the two cold plate branches are, respectively, 30 kg/h and 41 kg/h; after reaching the stable state this results in the outlet temperature of the cold plate 1 branch and the cold plate 1 temperature being higher than cold plate 2. In stage two, the trends are also the same as in case 2, except for the different response speed and the values of the valve opening in the stable state. A comparison is given in the following section.

### 3.4. Comparison of the Three Cases

The control strategy at the local level for cold plate 1 in the three cases shows no difference; the control variable is the valve opening of cold plate 1 and the controlled variable is the outlet temperature of the cold plate. The difference is seen within the control strategy at the global level, and, therefore, the focal point of the comparison is the impact of the global control strategy on the system.

**Figure 14:**
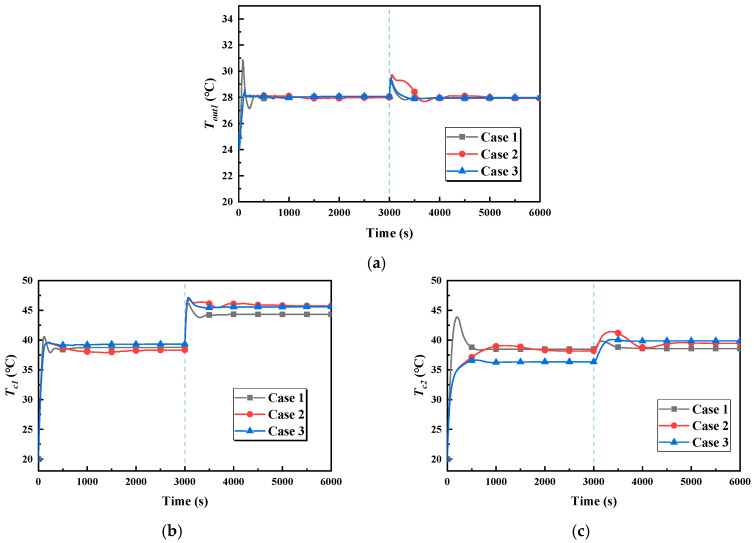
Temperature–time curves. (**a**) The outlet temperature of cold plate 1. (**b**) The temperature of cold plate 1. (**c**) The temperature of cold plate 2.

The outlet temperature of cold plate 1 is shown in Figure 14a. In stage one, the overshoot of case 1 is the biggest, while it kept within 1 °C in case 2 and case 3, and is 30.8 °C. However, the temperature is controlled within 1 °C in about 100 s and reaches the stable state in 400 s in case 1. In stage two, case 1 has the smallest overshoot and the shortest time to obtain a stable state, but with case 2 it is the opposite.

Figure 14b illustrates the variation in cold plate 1’s temperature in the three cases. In stage one, the temperature of cold plate 1 is both 38.5 ± 0.8 °C in the three cases, and in stage two it is both 45.5 ± 0.8 °C, which is the smallest difference observed. In all cases, the temperature of cold plate 1 does not exceed 50 °C, which meets the requirements. However, when comparing the stable temperature of the two stages, the temperature differences of the two stages in the three cases are 5.5 °C, 7.4 °C, and 6.3 °C, respectively. In terms of stable time, case 2 takes the longest.

Figure 14c illustrates the variation in cold plate 2’s temperature in the three cases. In stage one, the maximum value of the temperature is 43.8 °C at first in case 1, and the temperature reaches a stable state after 600 s. In the stable state, the temperatures are about 38 °C in both case 1 and case 2, while in case 3 the temperature 36.3 °C, which is the smallest stable temperature. In stage one, the temperatures are similar to one another, being 39 ± 0.8 °C. As for the temperature differences of the two stages in the stable state, in the three cases these are 0.2 °C, 1.3 °C, and 3.3 °C, respectively. It is obvious that the change in cold plate 2’s temperature is the biggest in case 3.

Taken together, without considering pump loss, case 1, in which the controlled variable is $T_{out}$ and the control variable is the pump speed, is the best choice. However, there are still situations in which the pump is not adjustable to reduced wear, and to prolong the service life of it, case 2, in which the controlled variable is $T_{out}$ and the control variable is $\varphi_{HX\prime }$, can be used. However, there is no denying that the response of the control strategy is slower than in case 1. Case 3, in which the controlled variable is $T_{in}$ and the control variable is $\varphi_{HX\prime }$, may not be considered because the regulation of the cold plate 1 branch has a big impact on the stability of cold plate 2’s temperature, although the heating power of cold plate 2 is constant all the time.

## 4. Conclusions

In this paper, an indirect coupling thermal control fluid loop system, along with intelligent control strategies, is proposed. The fluid loop system has an intermediate heat exchanger and an isolated fluid loop in the rack compared to the direct thermal control fluid loop, which is widely used in the ISS. Intelligent control strategies have been designed, along with a dynamic model, and three different cases which have different inputs and outputs of the controller are simulated. The results of the simulation have been compared and analyzed in detail. From the calculation results it can be seen that:

1. The overshoots are smaller and the settling times are shorter for the controlled variables with the fuzzy incremental controller compared to the PID controller. Thus, the fuzzy incremental controller is effective and is better than the traditional PID controller in the thermal control system.
2. As for the choice of the control and controlled variables, pump speed, and the outlet temperature of the cold plate branch, $T_{out}$ is the best choice because it has the fastest response and the most stable temperature of the three cold plates.
3. When considering the service life of the pump, the control and controlled variables can be the bypass valve opening of the intermediate heat exchanger and the outlet temperature of the cold plates branch $T_{out}$.
4. The control results are different in the three control strategies, but all of the cases reached the target temperature in an acceptable time and the temperatures of all the cold plates were below 50 °C, which demonstrates that the new indirect coupling thermal control fluid loop system is feasible for active thermal control of the experimental payloads in the rack.

The results show that an indirect coupling thermal control fluid loop system with fuzzy incremental controller can realize precise active thermal control of experimental payloads without adding complex assemblies. The fluid topology is widely applicable for the thermal control of scientific experimental racks in space stations, which is significant for the innovation of thermal control systems in the space stations.

## Figures and Tables

**Table 1:** Setting of control parameter at the local level.

Control Variable	Controlled Variable
Valve opening of each cold plate branch	$T_{out,i}$

**Table 2:** Setting of control parameters at the global level.

Number	Control Variable	Controlled Variable
1	Pump speed	$T_{in}$
2	Pump speed	$T_{out}$
3	Valve opening of the bypass of the heat exchanger	$T_{in}$
4	Valve opening of the bypass of the heat exchanger	$T_{out}$

**Table 3:** Fuzzy sets and linguistic values.

Fuzzy Sets	Ranks	Linguistic Values
PB	3	Positive big
PM	2	Positive medium
PS	1	Positive small
ZE	0	Zero
NS	−1	Negative small
NM	−2	Negative medium
NB	−3	Negative big

**Table 4:** Fuzzy decision rules.

$EC_{i}$	$E_{j}$						
	NB	NM	NS	ZE	PS	PM	PB
**NB**	PB	PB	PM	PM	PS	PS	ZE
**NM**	PB	PM	PM	PM	PS	PS	ZE
**NS**	PM	PM	PS	PS	PS	PS	NS
**ZE**	PS	PS	PS	ZE	NS	NS	NS
**PS**	PS	NS	NS	NS	NS	NM	NM
**PM**	ZE	NS	NS	NM	NM	NM	NB
**PB**	ZE	NS	NS	NM	NM	NB	NB

**Table 5:** Setting of control parameters of the global controller in three different cases.

Cases	Control Variable	Controlled Variable
1	Pump speed	$T_{out}$
2	Valve opening of the bypass of the heat exchanger	$T_{in}$
3	Valve opening of the bypass of the heat exchanger	$T_{out}$

**Table 6:** Main parameters used in the calculation.

Parameters	Values
Initial temperature	20 °C
Rack	
Length, width, and height	107×114×203 cm
Mass	400 kg
Pump	
Displacement	3 cc/rev
Typical speed *n*	150 rev/min
Cold plate	
Length, width, and height	20×20×1.8 cm
Mass	1 kg
Equivalent diameter	0.0101 mm
Pipe	
Hydraulic diameter	8 mm
Relative friction coefficient	0.00125
Primary water loop	
Inlet temperature	16 °C
Flowrate	175 kg/h

**Table 7:** The two stages of the simulation process.

Time	Stages	Heating Power of Cold Plate 1	Heating Power of Cold Plates 2 and 3
0–3000 s	One	250 W	250 W
3000–6000s	Two	500 W	250 W

**Table 8:** Comparison of the control effects of the fuzzy incremental and PID controllers.

Temperature	Controller	Stage One		Stage Two	
		Overshoots	Settling Time	Overshoots	Settling Time
***T_out_*_1_**	Fuzzy incremental	2 °C	325 s	1.2 °C	465 s
	PID	2.1 °C	400 s	1.4 °C	605 s
***T_out_***	Fuzzy incremental	1.7 °C	904 s	0.8 °C	413 s
	PID	2.4 °C	1175 s	1.1 °C	533 s

## References

[1] Joseph S., Neigut A., Judy M. (2017). International Space Station Facilities Research in Space 2017 and Beyond.

[2] NASA. https://www.nasa.gov/.

[3] Henshaw L.E., Sledd A. (1995). EXPRESS Rack-Space Station Subrack Payload Accommodations. SAE Tech. Pap..

[4] De Palo S., Wright B., Lake R., Challis S., Davenport R., Pietrafesa D. Updates on HRF Payloads Operations in Columbus ATCS. Proceedings of the 41st International Conference on Environmental Systems.

[5] Pelfrey J., Lee J. An EXPRESS Rack Overview and Support for Microgravity Research on the International Space Station (ISS). Proceedings of the 46th AIAA Aerospace Sciences Meeting and Exhibit.

[6] Sleiman A.Y. (1995). Active Thermal Control System Kit for International Standard Payload Rack. SAE Tech. Pap..

[7] Hartung R., Moore K.C. (1995). A Review of Space Station ECLSS/ITCS Automation. SAE Tech. Pap..

[8] Vaccaneo P., Gottero M. (2001). The thermal environmental control (TEC) of the fluid science laboratory (FSL): A combined (water/air) thermal design solution for a columbus active rack. SAE Tech. Pap..

[9] Patel V.P., Barido R., Johnson B., Ibarra T. (2001). Development of the internal thermal control system (ITCS) for international space station (ISS). SAE Tech. Pap..

[10] Sleiman A.Y., Rogers S.C. (1996). US Lab IATCS Hardware and Software Integration Development Test. SAE Tech. Pap..

[11] Wieland P.O., Roman M.C., Miller L. (2007). Living Together in Space: The International Space Station Internal Active Thermal Control System Issues and Solutions-Sustaining Engineering Activities at the Marshall Space Flight Center from 1998 to 2005.

[12] Valenzano G., Lombardi S., Prever E.B., Iannazzo S. (1998). ISS Node 2 TCS Design and Development. SAE Tech. Pap..

[13] Ray C.D., Perry J.L., Callahan D.M. (2000). International Space Station Sustaining Engineering: A Ground-Based Test Bed for Evaluating Integrated Environmental Control and Life Support System and Internal Thermal Control System Flight Performance. SAE Tech. Pap..

[14] Thurman R.L. (1994). Active Thermal Control of Parallel Heat Loads on Space Station US Laboratory Module. SAE Tech. Pap..

[15] Klingberg T. (2011). A Stability Analysis of the Active Thermal Control System of the Columbus Space Laboratory. Master’s Thesis.

[16] Valenzano G., Burzagli F., Lombardi S. (2001). Temperature Controller Stability Resolution for ISS Nodes 2& 3 IATCS Loops. SAE Tech. Pap..

[17] De P.S., Klingberg T., Persson J. (2009). Control Stability Analysis Applied to Columbus ATCS. SAE Tech. Pap..

[18] Guo W., Li Y., Li Y.Z., Zhong M.L., Wang S.N., Wang J.X., Zhang J.X. (2017). A self-driven temperature and flow rate co-adjustment mechanism based on Shape-Memory-Alloy (SMA) assembly for an adaptive thermal control coldplate module with on-orbit service characteristics. Appl. Therm. Eng..

[19] Lee S.H., Mudawar I., Hasan M.M. (2016). Thermal analysis of hybrid single-phase, two-phase and heat pump thermal control system (TCS) for future spacecraft. Appl. Therm. Eng..

[20] Stephan R. Overview of NASA’s thermal control system development for exploration project. Proceedings of the 40th International Conference on Environmental Systems.

[21] Kim T.Y., Hyun B.S., Lee J.J., Rhee J. (2013). Numerical study of the spacecraft thermal control hardware combining solid–liquid phase change material and a heat pipe. Aerosp. Sci. Technol. Aerosp. Sci. Technol..

[22] Cui Y., Zhang W., He B. (2016). A Variation-Aware Adaptive Fuzzy Control System for Thermal Management of Microprocessors. IEEE Trans. Very Large Scale Integr. (VLSI) Syst..

[23] Kwan T.H., Wu X., Yao Q. (2018). Integrated TEG-TEC and variable coolant flow rate controller for temperature control and energy harvesting. Energy.

[24] Li Y.Z., Lee K.M. (2011). Thermohydraulic Dynamics and Fuzzy Coordination Control of a Microchannel Cooling Network for Space Electronics. IEEE Trans. Ind. Electron.

[25] Zhao L., Man G.L., Cao J.F. (2011). Modeling and Simulation on Singe-phase Fluid Loop Control Algorithm. Spacecr. Eng..

[26] Jaber H., Webb R.L. (1989). Design of cooling towers by the effectiveness-NTU method. J. Heat Transf..

